# Pharmacokinetics of Piperaquine in Pregnant Women in Sudan with Uncomplicated *Plasmodium falciparum* Malaria

**DOI:** 10.4269/ajtmh.2012.11-0410

**Published:** 2012-07-01

**Authors:** Ishag Adam, Joel Tarning, Niklas Lindegardh, Hyder Mahgoub, Rose McGready, François Nosten

**Affiliations:** Faculty of Medicine, University of Khartoum, Khartoum, Sudan, Mahidol-Oxford Tropical Medicine Research Unit, Faculty of Tropical Medicine, Mahidol University, Bangkok, Thailand; Centre for Tropical Medicine, Churchill Hospital, Oxford, United Kingdom; New Hafa Teaching Hospital, New Halfa, Sudan; Shoklo Malaria Research Unit, Mae Sot, Tak, Thailand

## Abstract

The pharmacokinetic properties of piperaquine were investigated in 12 pregnant and 12 well-matched, non-pregnant women receiving a three-day oral fixed dose combination regimen of dihydroartemisinin and piperaquine for treatment of uncomplicated *Plasmodium falciparum* at New Halfa Hospital in eastern Sudan. Frequent venous plasma samples were drawn from the patients over a 63-day period and a complete concentration–time profile was collected for 7 pregnant and 11 non-pregnant patients. Piperaquine was quantified using a liquid chromatography–mass spectrometry/mass spectrometry method. Pregnant women had a significantly higher total drug exposure (median area under the curve [range] = 1,770 [1,200–5,600] hr × ng/mL versus 858 [325–2,370] hr × ng/mL; *P* = 0.018) and longer time to maximal concentration (4.00 [1.50–4.03] hr versus 1.50 [0.500–8.00] hr; *P* = 0.02) after the first dose compared with non-pregnant women. There was no other significant difference observed in piperaquine pharmacokinetics between pregnant and non-pregnant women, including no difference in total drug exposure or maximum concentration. The overall pharmacokinetic properties of piperaquine in this study were consistent with previously published reports in non-pregnant patients.

## Introduction

Malaria during pregnancy is a major public health problem in tropical and subtropical regions; each year 30.3 million African women become pregnant in malaria-endemic areas.^1^ Pregnant women are more susceptible to malaria than their non-pregnant counterparts.^2^,^3^ Malaria infections are associated with poor maternal and fetal outcomes, and malaria during pregnancy is a significant burden in Sudan^4^–^8^ and one of the leading causes of maternal mortality.^9^,^10^

Pregnant women infected with malaria should receive prompt treatment with effective and safe antimalarial drugs,^11^ a goal hampered by the spread of multidrug-resistant *Plasmodium falciparum* malaria in Sudan.^12^ After widespread malaria parasite resistance to antimalarials, artemisinin-based combination therapy has been introduced as first-line or second-line treatment for malaria in almost all malaria-endemic countries.^11^ Dihydroartemisinin-piperaquine (DHA-PQ) is a promising new fixed oral combination. Recent results from clinical trials show that DHA-PQ may be more effective against malaria in non-pregnant populations than the current widely used options, e.g., artemether-lumefantrine.^13^,^14^

A World Health Organization (WHO) expert committee concluded that artemisinins could be used during the second or third trimesters if no suitable alternative was available.^11^ However, pregnancy is associated with many physiological changes, which may have an impact on the pharmacokinetics of drugs.^15^,^16^ Thus, it is of value to study the pharmacokinetic properties of antimalarial drugs in this special group of patients to determine whether adjustment of dose or dose regimen is necessary. There is evidence that the pharmacokinetics of antimalarial drugs such as chloroquine, proguanil, atovaquone, artesunate, sulfadoxine, pyrimethamine, and lumefantrine are altered during pregnancy and doses used in non-pregnant patients may not be adequate in pregnant patients.^17^–^22^ Despite PQ use in pregnancy, as recommended by WHO, no studies on the pharmacokinetics of PQ in pregnancy in Africa have been published to date. Thus, the current study was conducted to investigate clinical and pharmacokinetic properties of PQ in pregnant and non-pregnant women in Sudan with uncomplicated *P. falciparum* malaria treated with DHA-PQ in their second and third trimesters of pregnancy.

## Materials And Methods

### Patients.

The study was conducted in New Halfa Hospital in the eastern Sudan during August 2007–February 2008. Twelve pregnant women with uncomplicated *P. falciparum* infections (with confirmed blood film of peripheral blood showing asexual forms of *P. falciparum*), but with no sign of severity,^23^ in the second or third trimester of gestation were recruited. Twelve age- and weight-matched non-pregnant women with uncomplicated *P. falciparum* malaria were recruited as controls.

Blood films were prepared and stained with Giemsa, and 100 oil-immersion fields were examined. The parasite density was evaluated by counting the number of asexual *P. falciparum* parasites for every 200 leukocytes, assuming a leukocyte count of 8,000 leukocytes/μL. All slides were double-checked in a blinded manner and only considered negative if no parasites were detected in 100 oil-immersion fields. Hemoglobin concentrations were estimated by using a HemoCue hemoglobin meter (HemoCue AB, Angelhom, Sweden).

After women signed an informed consent, relevant sociodemographic characteristics and clinical and obstetrics findings (age, parity, and gestational age) were collected from each patient by using pre-tested questionnaires. Inclusion criteria included a maternal age of 18–45 years, for pregnant women a gestational age of 15–40 weeks as determined by last menstrual period and confirmed by ultrasound examinations, absence of any chronic illness, and ability to follow the study schedule and provide written informed consent. Exclusion criteria included a history of taking any antimalarial in the previous 28 days, severe anemia (hemoglobin level < 7 g/dL), laboratory evidence of renal or hepatic impairment, or any other sign of severity.

### Treatment.

The drugs were administered with a glass of water after fasting conditions under the supervision as a fixed oral body weight–adjusted dose of DHA/PQ-phosphate (Duo-Cotecxin, Beijing, People's Republic of China) once a day for three days (i.e., 2.4 mg of DHA/kg body weight and 20 mg of PQ (as phosphate)/kg body weight per day) rounded to the nearest half tablet. Vomiting any of the treatment doses led to exclusion from the pharmacokinetic study.

### Ethical approval.

Each woman was explained in Arabic the procedures of the study and was given a written explanation that was read for her if she was unable to read by herself. She signed a written consent (or thumb-printed) before being admitted to the study. It was clearly explained that she could withdraw from the study at any time without adverse consequences. The study was approved by the National College for Medical and Technical Sciences, Sudan.

### Blood samples.

Blood samples were obtained by venous puncture and a three-way tap attached to the catheter. Blood (5 mL) was obtained before the first dose (day 0) and on day 14 for hematologic and biochemical tests and polymerase chain reaction (PCR).

Blood samples (2 mL) for pharmacokinetic analysis were collected at 0, 1.5, 4, 8, 24, 25.5, 28, 32, 48, 49, 50, 51, 52, 4, 56, 60, and 72 hours and then at days 5, 7, 14, 21, 28, 35, 42, 49, 56, and 63 after starting treatment. Blood samples were centrifuged at 2,000 × *g* for 10 minutes, and plasma was transferred into cryovials, stored in liquid nitrogen, and transferred to Khartoum where it was stored at –80°C until shipped on dry ice for drug analysis in Thailand. The same sampling schedule was applied to controls from pregnant and non-pregnant women.

The PQ plasma concentrations were determined by using solid-phase extraction and liquid chromatography coupled to tandem mass spectroscopy as described.^24^ Quality control samples at low, middle, and high concentrations were analyzed in triplicate within each analytical batch to ensure accuracy and precision during the analysis. Interassay precision was 4.2%, 2.7%, and 2.2% at 4.5, 20, and 400 ng/mL, respectively. The lower limit of quantification was set to 1.5 ng/mL.

### Clinical assessment and follow-up.

Patients (pregnant and non-pregnant women) were kept in the hospital for seven days and were seen daily; pregnant and non-pregnant women were then followed-up weekly in the antenatal and referral clinics, respectively. Daily evaluation (for the first seven days), including clinical (temperature, pulse, blood pressure) and parasitologic examinations, drug administration, and recording of side effects (on days 0, 1, 2, 3, and 7) in a case record form were performed. Women were screened for expected side effects daily, which included anorexia, nausea, diarrhea, vomiting, itching abdominal pain, joint pain, rash, skin pigmentation, dizziness, and tinnitus. Thereafter, the women were seen weekly. Parasitologic follow-up was continued for nine weeks or in some women until delivery. In case of re-appearance of *P. falciparum* parasites during the follow-up period, the patients received quinine, 10 mg/kg, three times a day for seven days and were followed-up weekly for four weeks. All women were requested to deliver at the hospital and data on outcome were recorded, which included sex, birth weight, duration of labor, and partogram. All newborns were examined by a pediatrician. Infants were seen monthly until one year of age and underwent neurologic developmental assessments at 3, 6, 9, and 12 months of age.

Low birth weight was defined as a birth weight < 2,500 g. A preterm delivery was defined as a gestational age between 28 and 37 weeks, and miscarriage was defined as delivery before 28 weeks.

An episode of *P. falciparum* malaria was regarded cured if peripheral blood of the women was free of parasites after treatment until day 63. In case of recrudescence within the follow-up period, the distinction between a re-infection and a true recrudescence was made by parasite genotyping with PCR as described previously.^25^

Clinical and biochemical data were compared between pregnant and non-pregnant women by using a Student *t*-test for normally distributed data and a non-parametric Mann-Whitney test for non-normally distributed data. A chi-square test was used to compare proportions between pregnant and non-pregnant groups. A *P* value < 0.05 was considered significant.

### Pharmacokinetic analysis.

Individual concentration–time data were evaluated by using a non-compartmental analysis approach in WinNonlin version 5.3 (Pharsight Corporation, Sunnyvale, CA). Residual PQ exposure from doses one and two cannot be accurately subtracted from the PQ exposure of the last dose (dose three) because of multi-compartment kinetics and a long terminal elimination half-life of approximately 20–30 days.^26^–^29^ Therefore, total amount of PQ base (three daily doses) was used as input dose with all observed concentration–time data in the non-compartmental analysis of PQ to compute pharmacokinetic parameter estimates. Total exposure up to the last measured concentration (AUC_0–LAST_) was calculated by using the linear trapezoidal method for ascending concentrations and the logarithmic trapezoidal method for descending concentrations. The PQ exposures during the 24-hour dose intervals were also calculated after each dose (AUC_0–24_, AUC_24–48_, AUC_48–72_). Drug exposure was extrapolated to time infinity by C_LAST_/λ_Z_ for each individual to compute total drug exposure (AUC_0–∞_) and total drug exposure in the post-treatment prophylactic phase (AUC_72–∞_). The terminal elimination half-life (t_1/2_) was estimated by log-linear regression of 3–9 observed concentrations in the terminal elimination phase. Maximum concentration (C_MAX_) and time to maximum concentration (T_MAX_) were taken directly from the observed data after each dose. Apparent volume of distribution (V_Z_/F) and oral clearance (CL/F) were computed individually according to standard procedures.

Mean pharmacokinetic parameter estimates were compared between the pregnant and non-pregnant women with malaria by using the Mann-Whitney test in GraphPad Prism 5 version 5.01 (GraphPad Software Inc., San Diego, CA). This test is a non-parametric test that compares the distributions of two unmatched groups. Parameter estimates were also compared with the result from previous studies reported in the literature.

## Results

### Clinical outcomes.

The two groups (pregnant and non-pregnant women) were well matched in their basic characteristics (Tables 1 and 2). The twelve pregnant women received the drug at the mean gestational age of 32.0 weeks (range = 15.0–40.0 weeks). By day 2, all patients (pregnant and non-pregnant) were febrile and their parasites had been cleared. Three patients had dizziness on the second and third days after treatment, two of these were non-pregnant and the third was pregnant. One of the non-pregnant women showed recurrent parasitemia on day 35. This finding was attributed to a new infection after PCR genotyping, which was performed on the same day. All other women were cured.

### Pregnancy outcomes.

There were no miscarriages in the study. There were three preterm deliveries with low birth weight babies. These three preterm deliveries occurred more than 90 days after receiving DHA-PQ. Overall, the range of the birth weight was 2,000–3,600 g (mean ± SD = 2, 891 ± 458 g. All babies were congenitally normal and no neurological deficit was observed by one year of age.

### Pharmacokinetics.

**Figure 1.**
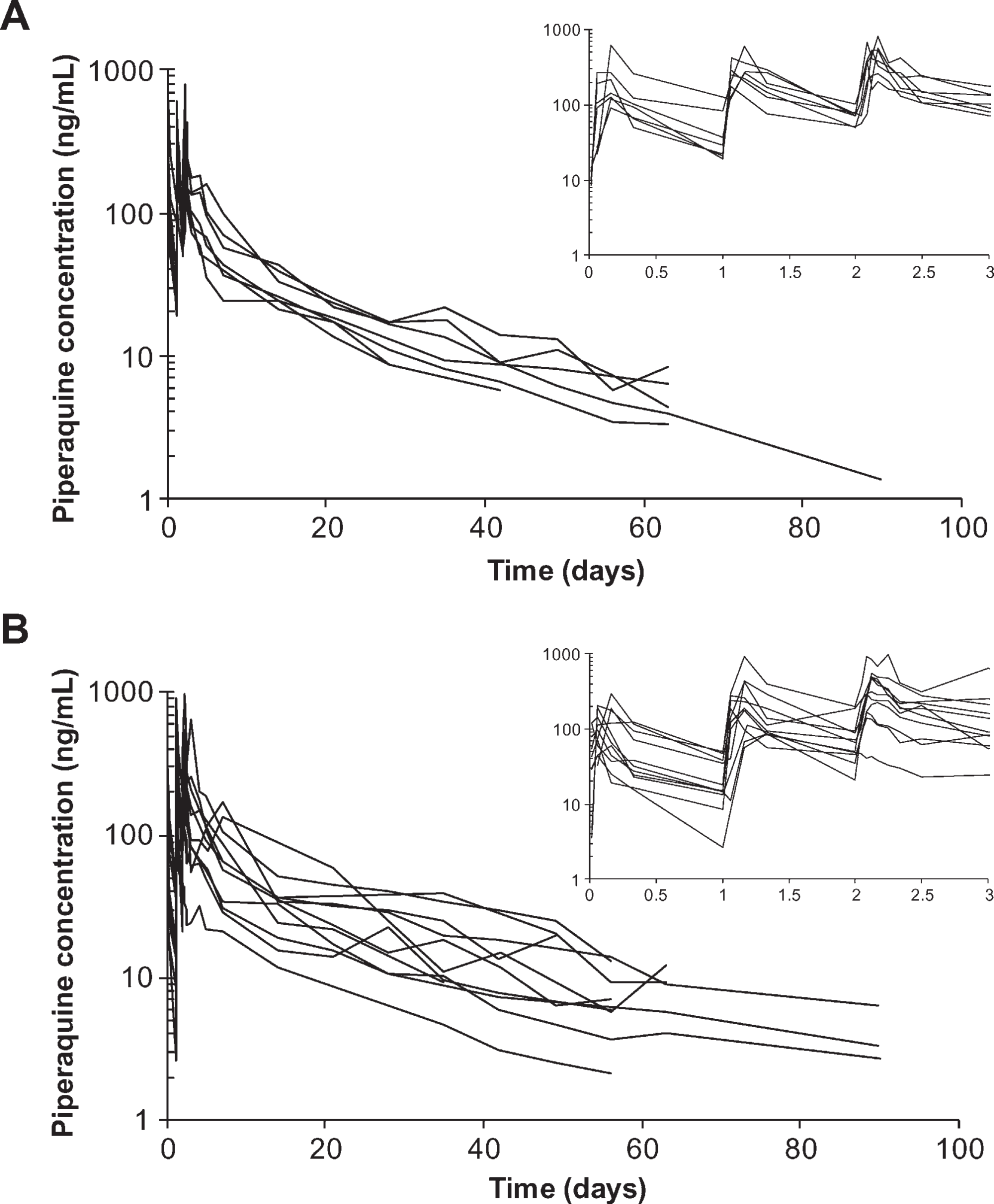
Piperaquine plasma concentration–time profiles in **A**, pregnant and **B**, non-pregnant women with uncomplicated *Plasmodium falciparum* malaria in Sudan.

The applied sampling schedule in general captured the pharmacokinetic profiles well in the studied population, and there was no sample below the level of PQ detection, i.e., 1.5 ng/mL (Figure 1

). Five patients in the pregnant group and one patient in the non-pregnant group were lost before three weeks of follow-up and were therefore excluded from the full pharmacokinetic analysis because they otherwise would have produced biased results because of the long half-life of the drug. However, these patients still contributed in the computation of pharmacokinetic parameters such as T_LAG_, T_MAX_, C_MAX_, and fractional AUC (i.e., AUC_0–24_, AUC_24–48_, and AUC_48–72_). Pregnant women had a significantly higher exposure (AUC_0–24_) and longer T_MAX_ after the first dose compared with non-pregnant women (Table 3) . There was no other significant difference in PQ pharmacokinetics between pregnant and non-pregnant women with uncomplicated *P. falciparum* malaria (Table 3). There was a trend of higher maximal PQ concentrations and a shorter half-life in pregnant women than in non-pregnant women but these differences did not reach statistical significance (Table 3). The pharmacokinetic properties of PQ vary to a great extent between studies but the overall pharmacokinetic properties of PQ in this study were consistent with those of published reports in non-pregnant patients with malaria (Table 4) .

## Discussion

In this study, DHA-PQ was effective and well tolerated. We have recently observed DHA-PQ to be efficacious in non-pregnant populations in central Sudan.^13^ Previously, 50 Karen pregnant women received DHA-PQ after reappearance after seven days of quinine (± clindamycin) or artesunate (± clindamycin). The treatment was effective (PCR-adjusted cure rate = 92.2% at day 63) and well tolerated, and there was no evidence of toxicity for the mothers or the fetus.^30^ Two babies whose mothers received DHA-PQ in the third trimester were observed to have hernia and chromosomal abnormality but their abnormailities were not attributed to the treatment.^30^ An additional 104 treatments at delivery were reported from West Papua^31^ and in a separate analysis of 1,160 treatments DHA-PQ was associated with a significant reduction in congenital malaria with DHA-PQ treatment.^32^ Animal studies have shown PQ to be safe during pregnancy.^33^ The women in the current study received DHA-PQ in the second and third trimesters, which is beyond the period of organogenesis in humans. Generally WHO recommends the use of artemisinin-based combination therapy (short-course, three-day treatments) in the second and third trimesters of pregnancy.^11^ The preterm deliveries with associated low birth weight babies in this study cannot be attributed to the treatment because they occurred more than 90 days after receiving DHA-PQ treatment. A community-based study in the same area observed that 2 of 15 pregnant women receiving quinine for uncomplicated *P. falciparum* malaria had pre-term labor.^2^

The pharmacokinetics of PQ reported in this study showed no statistical difference in total drug exposure between pregnant and non-pregnant women with malaria, which is reassuring. However, caution is needed when interpreting pharmacokinetic results from small samples sizes with large inter-individual variation, and further studies in a larger population are warranted. This caution limits the ability of this study to resolve the question of whether DHA-PQ dose adjustment should be considered in pregnant women. There was a trend of a shorter terminal elimination half-life in pregnant women than in non-pregnant women in this study but this difference did not reach statistical significance (Table 3). The same difference was recently reported in a study of 24 pregnant and 24 non-pregnant women on the Thai-Burmese boarder. This finding could be of clinical importance in intermittent preventive treatment and should be investigated in larger series.

Previously published results indicate that the pharmacokinetic properties of dihydroartemisinin after oral administration of artesunate might be altered during pregnancy with resulting lower drug exposure.^19^,^34^ Lower dihydroartemisinin exposure was also recently confirmed in in pregnant patients receiving dihydroartemisinin-piperaquine. Thus, the overall pharmacokinetic properties of PQ in this study were consistent with previously published reports in non-pregnant populations and pharmacokinetic properties of PQ vary to a great extent between studies.^27^–^29^,^35^–^38^ In the last study^38^ the PK values for PQ were obtained from the same healthy persons associated with a bioequivalence study given two different formulations of DHA-PQ (Arterakine versus Artekin).

In conclusion, although this was a small study, the fixed oral combination of DHA-PQ is a safe and effective treatment for pregnant women with malaria. No significant pharmacokinetic differences between pregnant and non-pregnant women with malaria were reported in this study but further studies are needed in larger populations.

## Figures and Tables

**Table 1:** Baseline clinical and biochemical characteristics of study population at admission in eastern Sudan*

Characteristic	Non-pregnant women (n = 12)	Pregnant women (n = 12)	*P*
Age, years	24.5 (5.6)	26.2 (8.7)	0.56
Weight, kg	59.9 (1.2)	61.3 (10.1)	0.77
Height, cm	164.5 (7.6)	166 (7.2)	0.93
Gestational age, weeks	–	32.0 (15.0–40.0)	–
Hemoglobin, g/dL	10.0 (1.4)	9.0 (1.0)	0.05
Parasite count, rings/μL†	14,288 (1,170–65,000)	12,642 (1,716–89,700)	0.78
Urea, mg/dL	25.3 (1.2)	26.1 (1.5)	0.16
Asparate aminotransferase, IU	4.5 (1.8)	5.4 (2.4)	0.31
Alanine aminotransferase, IU	12.5 (6.0)	13.5 (4.7)	0.65

* Values are mean (SD) unless otherwise stated.

† Geometric mean (range).

**Table 2:** Biochemical characteristics of study population at day 14 in eastern Sudan*

Characteristic	Non-pregnant women (n = 12)	Pregnant women (n = 12)	*P*
Hemoglobin, g/dL	10.0 (1.3)	9.1 (0.9)	0.06
Urea, mg/dL	25.5 (1.1)	25.6 (1.4)	0.84
Asparate aminotransferase, IU	4.7 (1.8)	5.2 (2.1)	0.53
Alanine aminotransferase, I U	12.0 (4.8)	13.1 (4.0)	0.54

* Values are mean (SD).

**Table 3:** Non-compartmental analysis of piperaquine pharmacokinetics in pregnant and non-pregnant women with uncomplicated *Plasmodium falciparum* malaria in eastern Sudan*

Characteristic	Piperaquine, pregnant women (n = 12) median (range)	Piperaquine, non-pregnant women (n = 12) median (range)	*P*
Body-weight (kg)	59.0 (50.0–72.0)	53.0 (44.0–81.0)	0.54
Total dose (mg/kg)	30.8 (27.7–32.6)	28.9 (27.4–32.0)	0.12
T_LAG_ (hr)	0 (0–0.500)	0 (0–0.500)	0.35
T_MAX 1_ (hr)	4.00 (1.50–4.03)	1.50 (0.500–8.00)	0.02
T_MAX 2_ (hr)	4.00 (1.50–4.12)	4.00 (1.50–8.00)	0.53
T_MAX 3_ (hr)	3.00 (2.00–6.00)	3.00 (0–6.00)	0.19
C_MAX 1_ (ng/mL)	203 (90.9–628)	133 (44.4–290)	0.08
C_MAX 2_ (ng/mL)	293 (179–605)	189 (84.9–917)	0.11
C_MAX 3_ (ng/mL)	374 (225–807)	312 (48.9–976)	0.27
CL/F (L/hr)†	36.4 (31.5–84.8)	33.1 (17.9–154)	0.25
CL/F (L/hr/kg)†	0.728 (0.500–1.18)	0.663 (0.264–1.93)	0.25
V/F (L)†	26,600 (19,900–52,600)	20,500 (12,100–87,600)	0.55
V/F (L/kg)†	437 (321–731)	466 (222–1,090)	0.96
T_1/2_ (days)†	17.9 (11.1–29.0)	24.3 (14.6–33.5)	0.22
AUC_0–24_ (hr × ng/mL)	1,770 (1,200–5,600)	858 (325–2370)	0.01
AUC_24–48_ (hr × ng/mL)	3,740 (1,950–5,110)	2,370 (1,120–8,800)	0.15
AUC_48–72_ (hr × ng/mL)	4,310 (2,550–7,160)	4,250 (696–12,700)	0.97
AUC_72hr–∞_ (hr × ng/mL)†	30,800 (18,800–46,300)	35,100 (11500–82,500)	0.22
AUC_0–LAST_ (hr × ng/mL)†	32,400 (22,700–56,000)	37,500 (13300–91,600)	0.49
AUC_0–∞_ (hr × ng/mL)†	38,100 (26,100–61,600)	41,800 (14400–106,000)	0.34
AUC_0–∞_/dose† (hr × ng/mL/(mg/kg)	1,370 (849–2,000)	1,510 (518–3,780)	0.25
Ext. AUC (%)†	9.05 (1.26–15.0)	10.2 (5.88–25.2)	0.49
Day 7 concentration (ng/mL)	50.5 (24.4–106)	56.9 (20.8–168)	0.46
Day 14 concentration (ng/mL)	32.4 (20.8–43.0)	33.4 (11.8–51.9)	0.67

* T_LAG_ = observed lag-time to absorption; T_MAX_ = observed time after dose to reach maximum concentration after doses 1, 2, and 3; C_MAX_ = maximum observed plasma concentration after doses 1, 2 and 3; CL = elimination clearance; V = apparent volume of distribution; T_1/2_ = terminal elimination half-life; AUC_0–24_ = observed area under the plasma concentration-time curve from zero time to 24 hours (i.e., first dose); AUC_24–48_ = observed area under the plasma concentration-time curve from 24 hours to 48 hours (i.e., second dose); AUC_48–72_ = observed area under the plasma concentration-time curve from 48 hours to 72 hours (i.e., third dose); AUC_72–∞_ = observed area under the plasma concentration-time curve from 72 hours to infinity; AUC_0–LAST_ = observed area under the plasma concentration-time curve from zero time to last observed concentration; AUC_0–∞_ = predicted area under the plasma concentration-time curve after the last dose from zero time to infinity, Ext. AUC percentage of AUC_0–∞_ extrapolated from the last observation to infinity; F = oral bioavailability; Day 7 concentration = observed concentrations at day 7; and Day 14 concentration = observed concentrations at day 14.

† Median (range) values are based on 7 pregnant and 11 non-pregnant women.

**Table 4:** Pharmacokinetic properties of piperquine reported in different studies*

Persons	Age (year)	No. of patients	Mean total dose (mg/kg) as base amount	Total no. of samples	Duration of sampling (days)	Food intake during drug administration	Pharmaco- kinetic analysis	CL/F (L/h/kg)	V_Z_/F (L/kg)	t_1/2, z_ (day)	Reference
Pregnant patients	24 ± 6	7	31	192	63 (28–90)	NI	NCA	0.73	437	16	This study
Non-pregnant patients	26 ± 10	11	29	315	56 (35–90)	NI	NCA	0.66	433	23	This study
Non-pregnant Patients	3–55	98	31	469	63	Not controlled	Mixed effects	1.4	874	28	Tarning and others, 2008^27^
Non-pregnant patients	6.9 ± 1.4	22	11.8	330	42	Not controlled	Compartmental analysis	0.85	431	17	Karunajeewa and others, 2008^28^
Non-pregnant Patients	30 ± 13	38	32	213	35	Fasting	Mixed effects	0.90	574	23	Hung and others, 2004^29^
Non-pregnant Patients	2–10	47	35	132	35	Fasting	Mixed effects	1.85	614	14	Hung and others, 2004^29^
Healthy volunteers	31 ± 3.5	12	25	468	29	Fasting	Mixed effects	1.00	103	12	Roshammar and others, 2006^35^
Healthy volunteers	19–42	8	4.2	152	42	Fasting	NCA	1.14	716	20	Sim and others, 2005^36^
Healthy volunteers	20.9 ± 1.6	6	4.9	120	28	Fasting	NCA	1.07	748	20	Nguyen and others, 2008^37^
Healthy volunteers	20.9 ± 1.6	6	9.8	120	28	Fasting	NCA	0.74	525	21	Nguyen and others, 2008^37^
Healthy male volunteer	21.0 ± 2.7	24	9.3	432	28	Fasting	NCA	0.40	353	26	Chinh and others, 2009^38^
Healthy male volunteer	21.0 ± 2.7	24	9.3	432	28	Fasting	NCA	0.47	394	25	Chinh and others, 2009^38^
Healthy volunteers	19–42	8	4.2	152	42	High fat food	NCA	0.60	365	21	Sim and others, 2005^36^

* Age is given as median (range) or mean ± SD. Pharmacokinetic parameters (i.e., elimination clearance [CL/F], apparent volume of distribution [V_Z_/F], and terminal elimination half-life [t_1/2,z_]) are given as mean values. NCA = non-compartmental analysis, compartmental analysis individual compartmental modeling, and mixed effects nonlinear mixed-effects modeling.
